# Effect of a Sodium and Calcium DL-*β*-Hydroxybutyrate Salt in Healthy Adults

**DOI:** 10.1155/2018/9812806

**Published:** 2018-04-12

**Authors:** Tobias Fischer, Ulrike Och, Ira Klawon, Tim Och, Marianne Grüneberg, Manfred Fobker, Ursula Bordewick-Dell, Thorsten Marquardt

**Affiliations:** ^1^Department of Food, Nutrition, and Facilities, FH Münster-University of Applied Sciences Muenster, Corrensstraße 25, 48149 Muenster, Germany; ^2^Department of Pediatrics, University Hospital Muenster, Albert-Schweitzer-Campus 1, 48149 Muenster, Germany; ^3^Center of Laboratory Medicine, University Hospital Muenster, Albert-Schweitzer-Campus 1, 48149 Muenster, Germany

## Abstract

**Background:**

Ketone body therapy and supplementation are of high interest for several medical and nutritional fields. The intake of ketone bodies is often discussed in relation to rare metabolic diseases, such as multiple acyl-CoA dehydrogenase deficiency (MADD), that have no alternatives for treatment. Case reports showed positive results of therapy using ketone bodies. The number of ketone body salts offered on the wellness market is increasing steadily. More information on the kinetics of intake, safety, and tolerance of these products is needed.

**Methods:**

In a one-dose kinetic study, six healthy subjects received an intervention (0.5 g/kg bw) using a commercially available ketone body supplement. The supplement contained a mixture of sodium and calcium D-/L-*β*-hydroxybutyrate (*β*HB) as well as food additives. The blood samples drawn in the study were tested for concentrations of D-*β*HB, glucose, and electrolytes, and blood gas analyses were done. Data on sensory evaluation and observed side effects of the supplement were collected. The product also went through chemical food analysis.

**Results:**

The supplement led to a significant increase of D-*β*HB concentration in blood 2.5 and 3 h after oral intake (*p*=0.033; *p*=0.043). The first significant effect was measured after 2 h with a mean value of 0.598 ± 0.300 mmol/L at the peak, which was recorded at 2.5 h. Changes in serum electrolytes and BGA were largely unremarkable. Taking the supplement was not without side effects. One subject dropped out due to gastrointestinal symptoms and two others reported similar but milder problems.

**Conclusions:**

Intake of a combination of calcium and sodium D-/L-*β*HB salt shows a slow resorption with a moderate increase of D-*β*HB in serum levels. An influence of *β*HB salts on acid-base balance could not be excluded by this one-dose study. Excessive regular consumption without medical observation is not free of adverse effects. The tested product can therefore not be recommended unconditionally.

## 1. Introduction

Interest in the importance of ketone bodies has risen in the recent past. Ketone bodies are an alternative fuel produced in the liver, in a process referred to as ketogenesis, in the event of reduced availability of glucose [1]. Insulin inhibits ketogenesis as opposed to glucagon and epinephrine, which both stimulate this process [2–4]. The base material is acetyl-CoA which is derived from the *β*-oxidation of fatty acids. The basis of all three “ketone” bodies is acetoacetate. Acetoacetate can then either be reduced to beta-hydroxybutyrate (*β*HB), or acetone is generated by spontaneous decarboxylation of acetoacetate. Only acetoacetate and *β*HB are relevant for energy expenditure [5, 6]. The maximum amount of daily ketone body production in adults is 150 g [7]. Normal postprandial *β*HB serum levels are less than 0.1 mmol/L [8]. They increase to approximately 0.1-0.2 mmol/L after fasting overnight in healthy subjects [9]. The term ketosis describes an increased concentration of ketone bodies in blood. In clinical application, ketosis is often defined as a concentration of ketone bodies in the range of 2–7 mmol/L and 3–5 mmol/L in therapy [10, 11].

The human organism has two nutrition-related ways of reaching ketosis. The first is starvation and the second is a high fat and at the same time low carbohydrate (HFLC) diet which is also known as a ketogenic diet [8, 12]. Ketosis can also be reached by an energy deficit caused by prolonged exercise [13]. In all cases, the organism reacts by an increasing ketone body production because of the decreased availability of glucose and therefore making the alternative fuel, ketone bodies, necessary as brain fuel or as energy substrate for other tissues, especially muscle [1, 8]. The main difference between the two nutrition-related states of ketosis is that in starvation fat reserves are used for ketone body synthesis and in HFLC fat from daily nutrition [8, 12]. Aside from the nutritional trend of low-carb diets with a maximal *β*HB serum concentration of 0.4 mmol/L, ketone bodies and specific diet forms like the ketogenic diet are of high scientific interest [14, 15].

There are different types of clinically relevant ketogenic diets. The milder forms are the low glycaemic index diet (LGID) and the modified Atkins diet (MAD) where carbohydrate intake is limited to 15 grams per day. More stringent are the medium chain triglycerides (MCT) diet as well as the 3 : 1 and the 4 : 1 classical ketogenic diets. The ratio depicts the fat contents in relation to the sum of carbohydrates and proteins based on their weight [16]. A well-known problem of ketogenic diets is limited patient compliance [17]. The daily intake of high amounts of fat is not tasty and has negative effects on the quality of life. Carbohydrate restriction is often even more difficult to adhere to. Adverse effects, especially soon after starting the diet, are often recorded and lead to dropouts. Typical undesirable side effects of the traditional ketogenic diet are gastrointestinal discomfort, weight loss, and negative changes in lipid profiles [18]. The application of moderate (≤50 g carbohydrates per day) ketogenic diets for weight loss show partially different effects when compared to clinically applied ketogenic diets. Positive effects on blood lipids, blood pressure, and weight are reported in obese subjects [19]. There are various very low carbohydrate and ketogenic trend diets that are milder than medical diets because trend diets have a lower fat content. The palatability of food in medical or trend diets increases strongly with the introduction of more carbohydrates and protein to the daily nutrition [20, 21].

The ketogenic diet has proven to be an effective therapy for epilepsy, although not only the anticonvulsive effect is used in medicine. In addition, some inherited metabolic diseases of glucose uptake or metabolism, for example, the GLUT1 or pyruvate dehydrogenase deficiency, are treated using ketogenic diets to ensure sufficient energy supply [22].

In special severe metabolic diseases of *β*-oxidation, like multiple acyl-CoA dehydrogenase deficiency (MADD), a direct intake of ketone bodies for energy supply is necessary. Inducing ketogenesis using normal food products is not possible due to the impairment of fatty acid oxidation. The genetic defect in the electron transport flavoprotein or the electron transport flavoprotein oxyreductase causes a dysfunction of all acyl-CoA dehydrogenases in *β*-oxidation by the impaired oxidation of FADH_2_ to FAD [23]. For this disease, direct ketone body therapy using D,L-3-hydroxybutyrate sodium salt can be life-saving. Ketone body therapy in MADD is documented in some case reports. In treated patients, an increase of D-*β*HB in serum was detectable within one hour of intake and led to dramatic clinical improvements. After 2 and 9 months, MRI investigations showed a progressive decrease of leukodystrophy, a typical problem in MADD patients [24]. In cases of hypoglycemia caused by hyperinsulinisim, treatment with *β*HB as a supplement has been used without any adverse side effects and increased the serum concentration of D-*β*HB [25].

Good efficacy of ketogenic diets is clouded by poor practicability and the necessity of maintaining a constant ketosis during the day. Therapeutic levels can be achieved more easily through oral intake of *β*HB [10]. The direct oral intake of ketone body salts or acid, as described in some case reports, may not be totally free of health risks. Possible adverse side effects are cation overload or acidosis/alkalosis [26]. Newer publications on ketone body salts concentrate on their application in sports [27, 28]. However, available knowledge is still very limited. To manage potential problems, researchers are concentrating on synthesizing ketone body esters consisting of the primary ketone *β*HB and an alcohol, for example 1,3-butanediol [29].

Supplements containing ketone bodies have potential applications in cases of severe metabolic diseases, cancer, neurodegenerative disease, and many more. Apart from the medical use, such supplements are also of interest for lifestyle applications such as weight loss [30].

In an Internet search of the worldwide supplement market, a lot of products with *β*HB as the main ingredient are available since 2015. The first available product was a simple mixture of calcium and sodium *β*HB salt with an added flavor. There is currently no scientific opinion or direct testing published about products with a calcium and sodium *β*HB salt as the main ingredient. The aim of this study is to provide additional scientific information for an easily available ketone body salt mixture and discuss the potential benefit of such products in clinical application.

## 2. Materials and Methods

### 2.1. Subjects

A total of six healthy adult subjects (3 males and 3 females) aged between 18 and 57 years (40 ± 15.9 years) were selected for this study. The participants, 4–normal, 1–overweight, and 1–obese, had an average BMI of 25.44 ± 5.99 kg/m^2^. The criteria for inclusion were absence of metabolic diseases (like diabetes), not pregnant, medically healthy with a normal medical history, and no intake of drugs (excluding oral contraceptives) or nutritional supplements in the previous 30 days prior to the start of the study. Subjects were excluded if they suddenly got ill or consumed a restrictive diet, low carb, or ketogenic diet, in the 60 days before the day of testing. Recruitment was done by putting up a notice on a board at the University Hospital of Muenster. After filling in a precheck questionnaire, a short clinical examination was performed by a medical doctor. On the day of testing, those that had qualified for inclusion had to appear with an empty stomach. The intake of food and caloric beverages was not allowed for 12 hours, 24 hours for alcoholic beverages, before testing began. Black unsweetened coffee and water were allowed on the testing day. All subjects were informed in detail about the study and signed the consent before they participated in the study. The study was conducted in accordance with the Declaration of Helsinki, and the protocol was approved by the Ethics Committee of the Medical Association of Westfalen-Lippe and the University of Münster (project identification code: 2017-402-f-S).

### 2.2. Procedure

A single-center one-dose kinetic study was conducted at the Muenster University Hospital, Germany, in accordance with the guidelines of good clinical practice. The present design of a one-dose kinetic study does not require randomisation, placebo-control, and blinding of the medical staff or subjects. The subjects fasted from 6 pm on the day before the test until the test began at 8 am. After weighing, placing a venous access and drawing the first blood sample (*t*_0_), the subjects ingested the prepared test solution. The solution was prepared for each subject by using the formula 0.5 grams of *β*HB-salt supplement per kilogram body weight dissolved in 250 milliliters (8.45 fl oz) of water. This translated to 30–57.5 g of the supplement per subject depending on their bodyweight. The taste of the beverage was recorded in a sensory interview after intake. Blood samples were drawn every 30 minutes over a period of 5.5 hours. Throughout testing, subjects were free to drink mineral water without gas and black unsweetened coffee or tea as well as move within the building. Each subject recorded observed side effects in a predetermined protocol. The concentrations of *β*HB and glucose were determined; blood gas analyses (BGA) in a two-sample series was performed every 2.5 hours, and the content of the minerals Na^+^, K^+^, and Ca^2+^ in serum was quantified. A medical doctor was available all the time and monitored the course of the study.

### 2.3. Nutritional Supplement

The supplement, with the trade name KetoCaNa Orange, is produced by a manufacturer in the USA (Ketosports) and was purchased from an online shop in the Netherlands. The main ingredient is a combination of sodium and calcium *β*HB-salt (racemic mixture; D-/L-*β*HB). Additional ingredients are citric acid, natural flavor, and stevia as sweetener. The supplement facts are shown in Table 1. All calories in the product are derived from the contained ketones. The producer recommends a serving size of 19 g powder dissolved in 236 ml (8 fl oz) of cold water that can be consumed up to three times a day. The producer mentions that the product properties praised have not been approved by the Food and Drug Administration (FDA) and the product is not intended for use in diseases.

### 2.4. Sampling and Analysis

#### 2.4.1. Blood Sampling

At the start of study, a venous catheter was placed and the first blood sample was drawn. For further sampling, the catheter was flushed with physiological saline. To avoid dilution of the sample, five milliliters of blood were discarded up front each blood sample. In case of a blocked venous access, a new catheter was placed. The samples were immediately cooled at −20°C for analysis on the same day. Otherwise, storage temperature was −80°C.

#### 2.4.2. *β*HB, Glucose, and Minerals in Serum

Serum levels of D-*β*HB were determined by using an enzymatic assay kit produced by Sigma-Aldrich (St. Louis, USA). The kit is designed to produce a compound whose colorimetric intensity, determined at a wavelength of 450 nm, is proportional to the concentration of D-*β*HB. Glucose and mineral (K^+^, Na^+^, and Ca^2+^) content in serum were determined using the analyzer Cobas 8000 manufactured by Roche Diagnostics (Mannheim, Germany) and carried out in the clinical laboratory of the university hospital in Muenster.

#### 2.4.3. Blood Gas Analyses

Blood samples were transported in a cooling box to the central laboratory immediately after sampling. The fully automated blood gas analyzer ABL800 Flex manufactured by Radiometer (Krefeld, Germany) was used to measure pH, electrolytes, and metabolites.

#### 2.4.4. Food Chemistry Analysis

Content of citric acid was determined using an enzymatic test kit for food, manufactured by Boehringer Mannheim (Darmstadt, Germany). The amount of oxidized NADH is stoichiometric to the citrate content. Difference between NADH and NAD^+^ was determined photometrically at 340 nm. Determination of pH was done using a pH meter and an electrode manufactured by Mettler Toledo (Giessen, Germany), after performing a three-point calibration. The quantification of minerals was carried out by a certified food laboratory. For sodium, potassium, calcium, and magnesium, ICP-MS and ICP-OES according to DIN EN ISO 11885/DIN EN ISO 17294-2 were used.

#### 2.4.5. Sensory Interview

The taste of the beverage was recorded in a face-to-face interview. All subjects had to describe the flavor and mouth-feel of the product in their own words supported. The questions of the interview were “Please describe as accurately as possible the taste of the beverage” followed by “How is the mouth-feel?” and “How would you describe the aftertaste?.” The last question was “Did you notice anything else?.” The test person was not to be interrupted when answering the questions, and the interviewer was prompted to interrupt only when responses were ambiguous or it was unclear what the test person meant. Especially positive or negative statements were examined more closely. The interview was recorded in its entirety.

### 2.5. Statistical Analysis

Data preparation was performed by using Microsoft Excel 2016, and for the data analysis, IBM SPSS Statistics 24 was used. Kolmogorov–Smirnov test, Shapiro–Wilk test, and graphical analyses were used to evaluate normal distribution. All data were first analyzed using descriptive statistics such as mean value, median, and standard deviation.

The basis for comparison was the first blood sample drawn from the subjects (*t*_0_). To calculate the difference between the results, a *t*-test for paired groups or Wilcoxon test was used. The level of significance was set at *p* ≤ 0.05. For relation among different sample series, the area under curve (AUC) was calculated.

In order to detect a clinical relevant change in the main outcome variable *β*HB (*μ*_1_ = 0.2; *μ*_2_ = 0.5; SD = 0.2; effect size dz = 1.5) with power of 80% and two-sided alpha of 0.05, a total of 5 participants were required. For security in case of dropout, one more subject (20%) was recruited.

## 3. Results

One of the subjects (female, obese) dropped out directly after imbibing the solution due to a severe reaction to the intake. These reactions included severe vomiting, nausea, and upper abdominal pain. All other volunteers (*n*=5) successfully completed testing.

### 3.1. *β*HB and Glucose Levels

**Figure 1:**
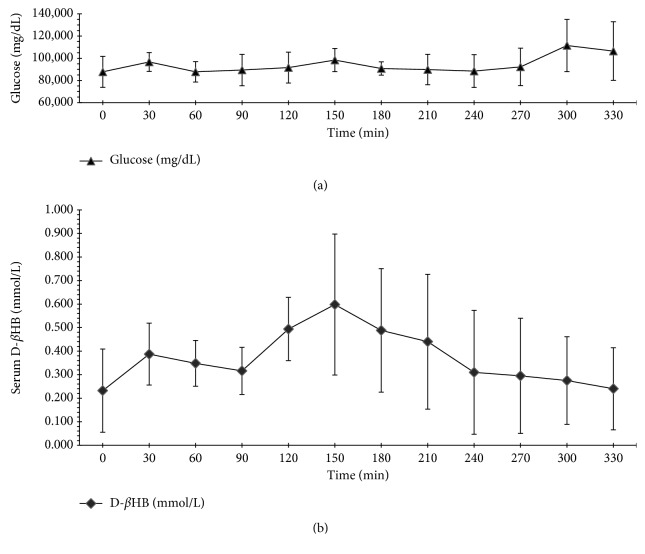
(a) Mean, standard deviation of blood glucose (mg/dL) and (b) mean, standard deviation of D-*β*HB (mmol/L) level in serum of all subjects (*n*=5) within a time period of 5.5 h after intake of *β*HB salt mixture (0.5 g/kg BW).

The 0.5 g/kg BW dose corresponded to 0.31 g D-/L-*β*HB/kg BW (3 mmol D-/L-*β*HB/kg BW) was calculated using the information provided by the manufacturer. The free D-*β*HB in serum increased after 2.5 hours from 0.232 ± 0.177 mmol/L at *t*_0_ to the maximal mean value of 0.598 ± 0.300 mmol/L at *t*_6_. The differences between 0 min, 150 min (maximum), and 180 min after treatment were significant (*p*=0.033; *p*=0.043). The first effect of the *β*HB salt intake was noted 2 hours later in the form of an increased concentration of serum D-*β*HB. Directly after reaching a maximum, the concentration declined continuously to near the baseline. There were large differences in D-*β*HB concentrations between the test persons as depicted in the rather large standard deviation in AUC (1.917 ± 0.811; *n*=4). Serum glucose concentrations in the serum of the test subjects remained constant almost right through the study period with an increase at the maximum of D-*β*HB concentration and after 4-5 hours. Both differences in serum glucose were not significant (*t*_5_*p*=0.741; *t*_10_*p*=0.689; Figure 1).

### 3.2. BGA and Serum Minerals

Blood gas analysis showed no difference in pH, pCO_2_, pO_2_, cations, and lactate concentration. An increase was detected in base excess (cBase; 2.350 ± 1.909 to 5.450 ± 0.071 mmol/L) and anion gap (1.450 ± 0.778 to 2.350 ± 1.061 mmol/L). The measured electrolytes sodium and calcium presented no difference during the study period. Potassium increased, and the increase was directly proportional to D-*β*HB levels in tested subjects. Determination of significance was not possible due to the small number of cases.

### 3.3. Side Effects

One of the subjects dropped out directly after solution intake due to severe vomiting, nausea, and upper abdominal pain. All other volunteers (*n*=5) finished the test successfully. One proband reported feeling of fullness directly after beverage intake, which was caused by the volume of the solution. Two others had nausea and slightly upset stomach in the first 30 minutes of testing. One of the subjects developed stomach cramps, diarrhea, and severe nausea after one hour, and a metoclopramide medication was necessary. In further course, one person felt hyperactive and three reported not feeling hungry over the complete study period.

### 3.4. Sensory Tasting

All subjects described the fragrance of the product as fruity and appetizing. One participant found the aroma unnatural and was reminded of medicine. The optical impression was neutral as the beverage had no color and was clear in appearance. Two test subjects found a color, corresponding to the fruit aroma, missing. One person reported a tolerable sour taste and four an extensive sour taste after solution intake. The flavor was pushed to the background by the high acidity. One subject additionally described the beverage as salty and soapy. Overall, the testers described the product as not being tasty and hard to drink especially in such a high volume.

### 3.5. Food Chemistry

Determination of citric acid showed a content of 0.286 g/g amounting to 5.434 g per serving size (19 g) with a mean pH of 4.32 when prepared according to the recommendation made by the manufacturer. An intake of 0.5 g/kg of the product corresponds to 0.7 mmol/kg citric acid. The content of minerals was analogous to the specifications on the product package. For magnesium and potassium, possible contaminations causing slight deviation were detectable (Table 2). The chosen dose of 0.5 g/kg in this study amounts to 1.3 mmol/kg sodium and 0.8 mmol/kg calcium. A three-times daily supplement intake of 0.5 g/kg would lead to an intake of 0.44 g/kg (2.2 mmol/kg) citric acid, 0.09 g/kg (3.9 mmol/kg) sodium, and 0.09 g/kg (2.3 mmol/kg) calcium.

## 4. Discussion

The results show a slow and moderate increase of D-*β*HB serum levels with a slow decline in healthy humans. Intake of a high concentration of D-/L-*β*HB supplement caused an average maximum increase of 0.366 mmol/L. Interpersonal variation of ketone body levels in humans is a well-known fact, a publication from 1958 determined a difference of 30 percent in healthy young men after fasting overnight [31]. In a collection of normal weight and overweight subjects, the standard deviation of *β*HB concentration was around 50 percent which is similar to our findings [32].

A direct comparison with other data is not possible due to absence of publications on the mixture of sodium and calcium D-/L-*β*HB salt. Only some case reports on the usage of sodium D-/L-*β*HB in severe metabolic diseases in children and studies testing different sodium and potassium D-/L-*β*HB salts are available. Van Hove et al. described a peak between 0.19 mmol/L and 0.36 mmol/L after 30 minutes to 1 hour caused by intake of 0.150 g/kg BW D-/L-*β*HB [24]. Gautschi et al. found a D-*β*HB concentration increase of 0.055 mmol/L within 1-2 h (150 mg/kg BW) and a measured maximum of 0.343 mmol/L after 2 h (200 mg/kg BW) [33]. Another group did not observe any rise in D-*β*HB concentration after the intake of 0.9 g/kg BW, and only after increasing the intake to 2.6 g/kg BW was there a measurable change in D-*β*HB levels [34]. All presented data originated from children with MADD and cannot be transfered to healthy adult subjects. There was an almost 10-fold increase in blood D-*β*HB within 0.5–3 h measured in two children with hyperinsulinism [25]. Especially, the second case report from Gautschi et al. shows in part a similar absorption of *β*HB salt when compared to our data [33]. In all case reports with children, no adverse effects were reported after the intake of sodium *β*HB [24, 25, 33, 34].

Testing of a ketone body ester consisting of D-1,3-butanediol and D-*β*HB (3-hydroxybutyl-3-hydroxybutyrate) exhibited a concentration peak between 1,5 and 2.5 h into testing. The recorded *c*_max_ D-*β*HB was 1.00 mmol/L achieved using 357 mg/kg BW of ester; this value corresponds to 2.80 mmol/L achieved using 1 g/kg BW of ester. In the single intake study, no adverse effects using a maximum dose of 714 mg/kg BW were reported. Only in a repeated dose study with a concentration of 2142 mg/kg BW per day did gastrointestinal side effects like vomiting, nausea, diarrhea, or abdominal pain occur [35]. A study with male athletes as test subject showed a rapid rise of D-*β*HB concentration in 10 minutes after drinking a solution made using 573 mg/kg BW of the 3-hydroxybutyl l-3-hydroxybutyrate ketone ester. In different experimental setups with young athletes, no adverse effects of *β*HB were reported [36]. In healthy subjects, Stubbs et al. showed a maximum increase of D-*β*HB concentration after 1.5 h to 1.00 ± 0.1 mmol/L after the intake of 282 mg/kg BW of a sodium and potassium D-/L-*β*HB salt. At the same time, tested *β*HB-ester leads to higher values at the same concentration (2.8 ± 0.2 mmol/L) [36]. Two further studies evaluated the adminstration of supplements containing D-/L-*β*HB salts to athletes during exercise. The levels of D-*β*HB concentration were between 0.60 and 1.00 mmol/L after the intake of the supplements [27, 28]. In all studies, no direct side effects were reported in healthy adults [27, 28, 37]. One publication reported a potential risk of gastrointestinal distress for high doses of *β*HB [27]. The tested product showed some gastrointestinal side effects directly after or within 2 h of solution intake. In comparison to these data, the intake of *β*HB was not high enough for specific side effects caused by ketone bodies. A possible reason is the high content of cations and citric acid in this product, causing the intense sour taste. As a whole, use of combination of calcium and sodium *β*HB salt is not free from adverse effects and exhibits a slow resorption kinetic. Just like in other supplements, such as the ester of 1,3-butandiol and *β*HB, the combined sodium and calcium salt exhibits a fast decrease in serum concentration. A direct comparison between the salts and the ester is not possible due to the structural differences and the metabolization of 1,3-butanediol being unclear. There are indications that 1,3-butandiol is metabolized to ketone bodies. This and the hydrolysis to D-*β*HB, not D-/L-*β*HB as in the salts, are possible explanations for the reported high *β*HB concentration in subjects after intake of this ester [35, 38, 39]. The sodium and potassium salt exhibits an earlier increase of D-*β*HB concentration in serum and a higher maximum using nearly the same dose when compared to our testing [37]. Likewise, the ester compound predominantly showed a faster increase of serum D-*β*HB concentration [35, 37, 40]. It seems that the salt combination affected the resorption of *β*HB. The results of athletes during exercise are not comparable to our population [27, 28].

A limitation of the measurements is the usage of an enzymatic assay specific for clinically relevant D-*β*HB. D-*β*HB acts as the main energy substrate in fasting humans and is therefore of high therapeutic relevance [8]. L-*β*HB, acetoacetate, and acetone were not analyzed in this study. Accordingly, an underestimation of total ketone bodies is possible. The effect of active substrates is considered to be low because there was an intake of *β*HB without longer fasting time period and a resulting short-time increase of *β*HB in serum. L-*β*HB is the nonphysiological enantiomer of D-*β*HB. There is an indication that L-*β*HB can be converted to physiological active ketone bodies (acetoacetate and D-*β*HB) and lipids in animal models but only in a limited amount [41, 42]. In humans, L-*β*HB showed a much lower metabolic rate and conspicuously higher elimination in urine than the D-*β*HB after intake of a D-/L-*β*HB salt [37]. More research is necessary to get more information about the metabolization of L-*β*HB in humans.

A one dose intake did not result in an increase of calcium and sodium. The relatively high amounts of both salts had no direct effect on their serum levels. In an example, a person weighing 70 kg would take around 2.100 g (91 mmol) sodium and 2.170 g (54 mmol) calcium with one dose (0.5 g/kg) of the supplement. The three-times daily dosage of 19 g product recommended by the manufacturer would lead to an intake of 3.420 g (149 mmol) sodium and 3.534 g (88 mmol) calcium. The calculated loads of both elements are above the tolerable upper intake level (UL) for adults recommended by the Institute of Medicine (IOM) (sodium 2.3 g/d (100 mmol/d); calcium 2.5 g/d (62 mmol/d)) [43, 44]. In comparison to the physiological need of sodium for body function, estimated at 500 mg/d (∼22 mmol/d), an estimated ∼4–7-fold increased ingestion takes place [45]. The high intake of sodium is combined with numerous adverse effects like thirst, fluid retention, hypertension, and higher risk for cardiovascular disease. Furthermore, a high sodium load has an effect on calcium metabolism expressed by higher calcium loss with a potential risk for urinary tract stones and bone demineralization [46–48]. An excessive ingestion of calcium leads to gastrointestinal side effects, renal stones, and a potential increase in cardiovascular events [49, 50]. The impact on cardiovascular diseases is still under discussion [50]. In case of an overload of sodium and calcium, a cumulative effect cannot be excluded. Long-term intake of high dosage of both elements could lead to side effects and have to be investigated further.

The finding of a similar increase between D-*β*HB and potassium levels needs further research. In our testing, we can exclude the influence of the test product (16 mg K^+^/100 g) or a pseudo hyperkalaemia by venous catheter.

The excessive intake of ketone body salt or acid could have consequences for the acid-base balance. According to a theory by Stewart, a shift of the strong ion difference (SID) can cause alkalosis or acidosis. On one hand, a high SID generated by intensive resorption of cations (e.g., Na^+^ and Ca^2+^) is a potential risk factor for alkalosis. On the other hand, a low SID and at the same time high strong ion gap (SIG) triggered by ketoacids can cause a metabolic acidosis [51, 52]. Stubbs et al. detected an increase of pH after intake of a sodium and potassium D-/L-*β*HB mixture, but no similar effect in a corresponding test using *β*HB-ester. Both products had a mild influence to the acid-base balance [37]. In an earlier study in the 1980s, a rise in pH after ketone body salt infusion was detected [53]. Surveillance of blood parameters, like the pH, pCO_2_, and product specific cations, are necessary to avoid adverse metabolic effects.

There are suggestions in the literature of an impact of increased *β*HB on reducing food intake. The mechanisms for this are not clear [54]. In our study, the majority of participants reported a loss of appetite and hunger beyond the testing time of 5.5 h. Thus, *β*HB may have an effect on the regulation of hunger and satiety. Further research with a double-blinded test design is needed to eliminate the influence of a known product ingredient.

In conclusion, the tested product cannot be recommended for an intake of 0.5 g/kg BW and leads to adverse gastrointestinal effects. The high acidity and general taste were not well accepted by the subjects making the product unsuitable for long-term application. Clinically, the high content of sodium, calcium, and the release of potassium is crucial and requires further research to establish short-term as well as long-term effects on the human organism. An additional aspect is that a higher intake is necessary to reach a ketosis sufficient for therapeutic purposes. Thus, an intake of 1 g/kg BW of the product would lead to a maximum of, in average, around 1.2 mmol/L (0.598 mmol/L at 0.5 g/kg BW). No interpersonal factors have been included in this simple calculation of our results. In combination with a higher intake, the cation load and proportion of additives increases, therefore increasing the potential risk of side effects. For treatment with high doses of external ketone bodies, further clinical trials on healthy adults are needed to gather more information on metabolic utilization.

## Figures and Tables

**Table 1:** Nutritional information and caloric content per serving size (19 g) and in 100 grams of the supplement.

	19 g (serving size)	100 g
Calories	68	358
*Macronutrients and minerals*	*Grams*	*Grams*
Fat	0	0
Carbohydrate	0	0
Protein	0	0
Sodium	1.30	6.84
Calcium	1.15	6.05
*β*HB	11.70	61.57

**Table 2:** Measured content in grams of selected minerals per serving size (19 g) and in 100 grams of the supplement.

Minerals	19 g (serving size)	100 g
Sodium (g)	1.140	6.000
Potassium (g)	0.003	0.016
Magnesium (g)	0.006	0.031
Calcium (g)	1.178	6.200
